# Effect of Preemptive Multimodal Analgesia Regimen on Post-operative Epidural Demand Boluses in Lower Limb Orthopaedic Surgeries

**DOI:** 10.7759/cureus.33958

**Published:** 2023-01-19

**Authors:** Mathew George, Kiran N, Ravi M

**Affiliations:** 1 Anaesthesiology, Sri Devaraj Urs Medical College, Sri Devaraj Urs Academy of Higher Education and Research, Kolar, IND

**Keywords:** fracture, postoperative, lower limb, analgesia, multimodal, preemptive

## Abstract

Introduction

Excruciating pain is associated with lower limb orthopaedic surgeries involving femoral shaft fractures. Postoperative pain management is still ineffective in low-resource settings where the use of epidural and opioid-free analgesia is impractical. Literature is scarce with respect to the effect of a preemptive multimodal analgesia regimen on the requirement of postoperative epidural demand boluses. Hence, the present study aimed to evaluate the effect of pre-emptive multimodal analgesia in reducing the requirement of epidural demand boluses postoperatively, and to find out the time required to receive the first epidural bolus.

Material and methods

This double-blinded randomized control study included 48 subjects. Patients aged 18-60 years with lower limb fractures requiring surgery under combined spinal-epidural anesthesia were included. Patients were divided into two groups through random allocation. Group A: Preemptive multimodal group received intravenous paracetamol 1 g, IV diclofenac 75 mg diluted in 100ml NS, IV tramadol 50 mg diluted in 100ml NS and tab pregabalin 75 mg orally, 30 mins before surgery. Group B: Placebo group received 3 pints of 100ml NS IV and tab ranitidine 150 mg, 30 mins before surgery. Intraoperatively, combined spinal-epidural anaesthesia was administered taking all the aseptic precautions. Visual analogue scale (VAS) was recorded immediately on shifting to a postoperative room, and then at 1, 4, 8, 12, and 24 hr for both groups. Epidural boluses (10 ml of 0.125% bupivacaine with 2 μg/ml of fentanyl) were given whenever the patient’s visual analogue scale was more than 4. The time at which the first epidural bolus was required by the patient was recorded. The total number of epidural boluses given over 24 hours based on VAS was recorded for both, the preemptive and placebo groups. If the patient still complained of pain, IV diclofenac 75 mg was given if the VAS was more than 4, while IV diclofenac 75 mg along with IV tramadol 50 mg was given if the VAS was more than 6. Patient satisfaction with anesthesia care, in general, was assessed 24 hrs postoperatively.

Results

A total of 48 subjects were included in the study. During the immediate-postoperative period, and at 8, 12 and 24 hr, the median VAS was significantly low in group A as compared to group B. A significant increase in the demand for epidural bolus immediate-postoperatively was observed in group B (70.83%) compared to group A (4.17%) (p-value of <0.001). At 8 hr, 12hr, and 24hr, patients in group A found a significantly less need for epidural boluses compared to Group B. The mean total number of epidural boluses taken in group A was significantly less compared to group B (1.79 ± 0.41 VS 3.33 ± 0.48, p-Value <0.001). In group A, all patients reported no requirement for diclofenac and tramadol. In group B, 8.33% required diclofenac 75 mg, while the remaining 91.66% had no requirement for diclofenac and tramadol. The difference in patient satisfaction with anaesthesia care in general between the two study groups was found to be significant with a p-value of 0.027. Patients in Group A were very satisfied compared with those in group B.

Conclusions

The study found that the pre-emptive multimodal analgesia group had better postoperative pain control because they required fewer epidural boluses and no extra analgesics postoperatively. This group was more satisfied with the anaesthesia care in general.

## Introduction

Preemptive administration of analgesic medication is considered to be more effective in controlling postoperative pain than medication given after the onset of a painful stimulus [1]. Previous studies with preemptive analgesia show evidence of reduced postoperative opioid requirement [2]. Multimodal analgesia combines analgesic medications from different classes and employs analgesic techniques that target various mechanisms of pain [3]. This is recommended in the treatment of acute postoperative and trauma-related pain because its synergistic effect maximizes pain relief at lower analgesic doses and hence, reduces the risk of adverse drug reactions [4]. The combined spinal-epidural approach combines the advantages of spinal block with the versatility of an indwelling epidural catheter for sustained analgesia further to the postoperative period by injecting a low dose of subarachnoid local anaesthetic, and then extending the block by injecting drug through the epidural catheter [5].

Lower limb orthopaedic surgeries such as fracture shaft of the femur are linked with excruciating pain. These operations are more often managed under combined spinal-epidural anaesthesia. Poorly managed pain can lead to nausea, vomiting, ileus, delayed feeding, and immobilization. Inadequate management of pain increases the rate of postoperative morbidity and mortality. Hence, the management of postoperative pain carries the utmost significance. Literature is scarce with respect to the effect of preemptive multimodal analgesia regimen on the requirement of postoperative epidural demand boluses [6]. The present study aimed to evaluate the effectiveness of preemptive multimodal analgesia in reducing the requirement of epidural demand boluses postoperatively, to find out the time required to receive the first epidural bolus, and to assess patient satisfaction in the preemptive group. This article was previously presented as a paper at the 31st Annual State Conference of Indian Society of Anesthesiologist - Andra Pradesh State ( ISACON AP ) on October 8, 2022.

## Materials and methods

After obtaining Institutional Ethics Committee approval (SDUMC/KLR/IEC/610/2020-21 dated 24/12/2020), we designed a double-blinded randomized controlled trial involving 48 patients undergoing lower limb orthopaedic surgery under combined spinal-epidural anaesthesia in Sri Devaraj Urs Medical College, Sri Devaraj Urs Academy of Higher Education and Research, Karnataka, India. Written, informed consent was obtained from all patients. Inclusion criteria in the study were: age over 18 years, American Society of Anesthesiologist’- physical status (ASA-PS) grades I and II, and patients posted for lower limb orthopaedic surgeries under combined spinal-epidural anaesthesia. The duration of recruitment was from January 2021 to May 2022. Patients unwilling to give informed consent, patients with known hypersensitivity to preemptive analgesic drugs, patients with an associated head injury, patients with renal impairment, polytrauma patients, patients with psychiatric disorders, and obese patients were excluded from the study. Randomization was done based on computer-generated randomization codes into two groups.

A detailed history of the patient was taken on the previous day of surgery. A complete physical examination was done. Routine investigations were checked. The intravenous line was secured and IV fluids were connected. Patients were divided into two groups based on computer-generated randomization. Sequentially numbered wrapped opaque envelopes that were given to the nurse in charge of the preoperative holding area for patients were used to conceal the randomization process. The anaesthesiologist selected a sealed envelope using the serial number label on the envelope and gave medications in accordance with the group regimen 30 mins prior to the scheduled surgery. Group A: Preemptive multimodal group received intravenous (IV) paracetamol 1 gm, IV diclofenac 75 mg diluted in 100ml NS, IV tramadol 50 mg diluted in 100ml NS, and tab pregabalin 75 mg orally, 30 mins before surgery. Group B: Placebo group received 3 pints of 100ml NS intravenously and tab ranitidine 150 mg orally, 30 mins before surgery. Tablets were given in a powdered form. The drug administered to the patient was unknown to them. Intraoperatively, combined spinal-epidural anaesthesia was administered under all aseptic precautions. Bupivacaine heavy of 3.4 cc was used for giving spinal anaestheisa. Visual analogue scale (VAS) was recorded immediate-postoperatively, and then at 1 hr, 4 hr, 8 hr, 12 hr, and 24 hr for both groups by another anaesthesiology resident. Immediate-postoperative corresponds to 2 hrs after giving spinal anaesthesia. Epidural bolus was given for postoperative pain management in both groups. Epidural boluses were given whenever the patient’s visual analogue scale was more than 4. An epidural bolus of 10 ml of 0.125% bupivacaine with 2 μg/ml of fentanyl was given. The time at which the first epidural bolus was required by the patient was recorded. The total number of epidural boluses given over 24 hours based on visual analogue scales had been recorded for both the preemptive and placebo groups. If the patient still complained of pain, IV diclofenac 75 mg was given if VAS was more than 4, IV diclofenac 75 mg along with IV tramadol 50 mg was given if VAS was more than 6. The requirement of IV diclofenac and IV tramadol was noted. Patient satisfaction with anesthesia care, in general, was assessed 24 hrs postoperatively using 4-point Likert scale (very satisfied/satisfied/dissatisfied/very dissatisfied). The 4-point Likert scale was taken from the Bauer questionnaire [7]. The patient was asked to give a reply based on their satisfaction and discomfort towards anaesthesia care. 

Statistical analysis

To detect a mean reduction of 1 in the number of epidural demand boluses among the preemptive multimodal analgesia group, considering an α error of 1% with the power of 90% and variance estimate of .81 in the number of epidural demand boluses as reported in a study by Makkar JK et.al., the estimated sample size was 24 per group [6]. For normally distributed quantitative parameters, the mean values were compared between study groups using independent sample t- test (2 groups). For non-normally distributed quantitative parameters, medians and interquartile range (IQR) were compared between study groups using the Mann-Whitney U test. The association between categorical explanatory variables and categorical outcomes was assessed by cross-tabulation and comparison of percentages. The Chi-square test was used to test statistical significance. P-value < 0.05 was considered statistically significant. Data was analysed by using coGuide software, V.1.01. BDSS Corp. Released 2020. coGuide Statistics software, Version 1.0, India: BDSS corp.

## Results

A total of 48 subjects were included in the final analysis. Among the study population, 24 (50%) participants were in group A, and the remaining 24 (50%) participants were in group B (Table 1).

**Table 1:** Comparison of age with study group in the study population (N=48) Independent sample t-test

Parameter	Study group		p-value
	Group A (N=24) Mean ± SD	Group B (N=24) Mean ± SD	
Age	42.46 ± 17.24	50.88 ± 19.98	0.1251

The mean age of group A was 42.46 ± 17.24 and group B was 50.88 ± 19.98, and the difference between the two groups was statistically not significant (p-value 0.1251). In group A, 21 (87.5%) were male, and the remaining 3 (12.5%) were female. In group B, 17 (70.83%) were male, and the remaining 7 (29.17%) were female. The difference in gender between the two groups was statistically not significant (P-value 0.2865). 

The median immediately-postoperative VAS of group A was 2 and group B was 5. The difference in VAS immediately postop between group A and group B was statistically significant. The median VAS after 1 hr was not significant between the two groups. Immediately postop, 70.83% of group B received epidural bolus while only 4.17% of group A received epidural bolus. Preemptive drugs were able to give superior analgesia. At 4hr, VAS was lower in group B due to the fact that most of the group B patients already received epidural bolus for postoperative pain management. The median VAS after 8hr, 12hr, and 24hr was lower in group A compared to Group B revealing that the effect of preemptive multimodal analgesia stayed longer (Table 2).

**Table 2:** Comparison of mean of VAS scores at different time periods between the study group (N=48) VAS - Visual analogue scale, Immediate post-operative corresponds to 2 hours after giving spinal anaesthesia Mann-Whitney U test

Parameter	Study group		Mann-Whitney U test p-value
	Group A (N=24) Median (IQR)	Group B (N=24) Median (IQR)	
VAS score during the immediate postoperative period	2.00 (2.0 to 3.0)	5.00 (3.0 to 5.25)	<0.001
VAS score after 1hr	3.00 (3.0 to 3.0)	3.00 (3.0 to 3.0)	0.2437
VAS score after 4hr	4.00 (2.75 to 4.0)	3.00 (3.0 to 3.0)	0.0347
VAS score after 8hr	3.00 (2.0 to 4.0)	5.00 (3.75 to 6.0)	<0.001
VAS score after 12hr	3.00 (3.0 to 3.0)	4.50 (3.0 to 6.0)	0.0023
VAS score after 24hr	4.00 (3.0 to 4.0)	5.00 (5.0 to 5.25)	<0.001v

The epidural bolus requirements immediate-postoperatively, and at 8hr, 12hr, and 24hr were significantly lower in group A compared to group B (Table 3).

**Table 3:** Comparison of epidural bolus immediate-postoperatively (2 hours after giving spinal anaesthesia), 1hr, 4hr, 8hr, 12hr, and 24hr with study group in the study population (N=48) Chi-square test

Epidural bolus requirement postoperatively	Study group		Chi-square value	p-value
	Group A (N=24)	Group B (N=24)		
Given immediate-postoperatively	1 (4.17%)	17 (70.83%)	22.76	<0.001
Not given immediate-postoperatively	23 (95.83%)	7 (29.17%)		
Epidural bolus after 1hr				
Given	2 (8.33%)	5 (20.83%)	1.51	0.4158
Not given	22 (91.67%)	19 (79.17%)		
Epidural bolus after 4hr				
Given	14 (58.33%)	2 (8.33%)	13.50	<0.001
Not given	10 (41.67%)	22 (91.67%)		
Epidural bolus after 8hr				
Given	7 (29.17%)	18 (75.00%)	10.10	0.0015
Not given	17 (70.83%)	6 (25.00%)		
Epidural bolus after 12 hr				
Given	5 (20.83%)	15 (62.50%)	8.57	0.0034
Not given	19 (79.17%)	9 (37.50%)		
Epidural bolus after 24 hr				
Given	14 (58.33%)	23 (95.83%)	9.55	0.0020
Not given	10 (41.67%)	1 (4.17%)		

The mean total number of epidural boluses of group A was 1.79 ± 0.41 and group B was 3.33 ± 0.48, and the difference in the total number of epidural boluses between group A and group B was statistically significant (p-value <0.001) (Table 4).

**Table 4:** Comparison of the total number of epidural boluses with study group in the study population (N=48) Independent sample t-test

Parameter	Study group		Independent sample t-test p-value
	Group A (N=24) Mean ± SD	Group B (N=24) Mean ± SD	
Total number of epidural boluses	1.79 ± 0.41	3.33 ± 0.48	<0.001

The difference in time at which the patients requested the 1st epidural bolus between the two study groups was found to be significant with a p-value of <0.001, where the majority of 17 (70.83%) participants demanded bolus during the immediate-postoperative period (Table 5).

**Table 5:** Comparison of time at which 1st epidural demand bolus with the study group in the study population (N=48) Chi-square test

Time at which 1st epidural bolus was requested	Study group		Chi-square value	p-value
	Group A (N=24)	Group B (N=24)		
Immediate post-operative	1 (4.17%)	17 (70.83%)		
1st hr	2 (8.33%)	5 (20.83%)	31.27	<0.001
4th hr	14 (58.33%)	1 (4.17%)		
8th hr	7 (29.17%)	1 (4.17%)		

In group B, diclofenac 75 mg was required for 2 patients (Table 6).

**Table 6:** Comparison of Requirement of diclofenac and tramadol with study group in the study population (N=48) Chi-square test

Requirement of diclofenac and tramadol	Study group	
	Group A (N=24)	Group B (N=24)
Diclofenac 75MG	0 (0.00%)	2 (8.33%)
Not required	24 (100%)	22 (91.66%)

The mean time at which the 1st epidural demand bolus was given (hr) in group A was 4.75 ± 2.40 and in group B it was 0.71 ± 1.78 (Table 7).

**Table 7:** Comparison of time at which the 1st epidural demand bolus was given with study group in the study population (N=48) Independent sample t-test

Parameter	Study group		IST p-Value
	Group A (N=24)	Group B (N=24)	
	Mean ± SD	Mean ± SD	
Time at which 1st epidural demand bolus given (hr.)	4.75 ± 2.40	0.71 ± 1.78	<0.001

Group A people were very satisfied compared with group B (Table 8). No patients in the preemptive multimodal group reported any side effects including drowsiness, dizziness, or somnolence. The incidence of postoperative nausea and vomiting appeared the same in both groups.

**Table 8:** Comparison of patient satisfaction with anaesthesia care in general between study groups (N=48) Chi-square test

Patient satisfaction with anaesthesia care in general	Study Group		Chi-square	p-value
	Group A (N=24)	Group B (N=24)		
Very Satisfied	10 (41.67%)	4 (16.67%)	9.143	0.027
Satisfied	10 (41.67%)	6 (25%)		
Dissatisfied	3 (12.5%)	11 (45.83%)		
Very Dissatisfied	1 (4.17%)	3 (12.5%)		

## Discussion

This study involved 48 subjects with 24 participants each in group A and group B. There was no significant difference in the age and proportion in gender between the two groups. 

The present study evaluated pain through VAS and found decreased pain scores in the MMA group. Makkar et al. [6] also evaluated pain using VAS. There was a significant difference in the requirement of epidural boluses between the preemptive and placebo group in both studies. The median VAS between both groups did not differ significantly. In group A, patients' median VAS was significantly lower immediate-postoperatively, 8hr, 12hr, and 24hr compared to group B. In a study by Passias B et al [8] using acetaminophen, celecoxib, and gabapentin administration 30-60 minutes before total joint arthroplasty resulted in modest reductions in opioid requirements postoperatively. 

Aweke et al. [9] used numerical pain scoring and found that the median NRS score was substantially lower in the paracetamol-tramadol group at the 4th, 6th and 8th hours compared to the paracetamol group. Our study also used paracetamol and tramadol along with other preemptive medications and found that median VAS pain score were lower in the preemptive group.

Preemptive 75 mg pregabalin for lower extremity orthopaedic surgeries was found to decrease postoperative pain, especially within the first 24 hours of surgery, and additionally reduced opioid consumption [10]. In addition, Omara et al. [11] discovered that oral pregabalin significantly sped up the time taken for the sensory block to two-segment regress and enhanced sleep quality the first night following surgery. Preoperative oral pregabalin improved sleep the first night following surgery and postponed the need for postoperative analgesics. Similarly, we also used pregabalin in our study and found acceptable results. Perioperative hemodynamics were within normal limits. Patients did not complain of any discomfort. In our study also, pregabalin was given along with paracetamol, tramadol, and diclofenac. This reduced the requirement for postoperative epidural demand boluses and the patients did not complain of nausea and vomiting. Reduced epidural requirement and more patient satisfaction were seen in the preemptive group.

The difference in patient satisfaction with anaesthesia care, in general, between the two study groups was found to be significant with a p-value of 0.027. There were more patients in the "very satisfied" category in group A as compared to group B. Similarly, in the study by Kheirabadi et al. and Sebastian et al. found an increased patient satisfaction score in the preemptive group [10,12].

According to a study done in Connecticut, multimodal analgesics only had fewer side effects like sedation, nausea, vomiting, pruritus, and constipation in addition to providing better pain relief. Studies had shown that combining multimodal analgesia with a rehabilitation program can result in a quicker recovery, a shorter stay in the hospital, and a shorter convalescence period [13]. In the present study, the preemptive multimodal analgesics were effective in the management of postoperative pain. Medications had the least side effects, patients expressed more satisfaction towards anaesthesia care, in general. 

Limitations

The current study has some limitations, including a small sample size, involvement of different anesthetists and surgeons and, the inability to control confounding factors like incision size. Our study did not compare intra-operative hemodynamics between the two groups. We did not record whether any analgesic drugs were administered to patients prior to their arrival in the preoperative room (patient-controlled epidural analgesia pumps could have been used).

## Conclusions

A preemptive multimodal analgesia regimen of IV paracetamol 1 g, IV diclofenac 75 mg, IV tramadol 50 mg, and tab pregabalin 75 mg orally, 30 mins before surgery reduced the requirement of epidural boluses, and prolonged the time required to receive the first epidural demand bolus compared to placebo in patients undergoing lower limb orthopaedic surgeries. VAS was significantly lower in the preemptive group. The analgesics combination seemed to be superior as patients were more satisfied. There was no requirement for additional analgesics in the preemptive group.
